# Canadian Healthcare System and Individuals with Severe Mental Disorders During Coronavirus Disease 2019: Challenges and Unmet Needs

**DOI:** 10.1093/schizbullopen/sgac036

**Published:** 2022-10-18

**Authors:** Leanna M W Lui, Roger S McIntyre

**Affiliations:** Institute of Medical Science, University of Toronto, Toronto, ON, Canada; Mood Disorders Psychopharmacology Unit, Poul Hansen Family Centre for Depression, University Health Network, Toronto, ON, Canada; Institute of Medical Science, University of Toronto, Toronto, ON, Canada; Mood Disorders Psychopharmacology Unit, Poul Hansen Family Centre for Depression, University Health Network, Toronto, ON, Canada; Department of Psychiatry, University of Toronto, Toronto, ON, Canada; Department of Pharmacology, University of Toronto, Toronto, ON, Canada

**Keywords:** COVID-19, healthcare, health systems, mood disorders, psychosis, Canada

## Abstract

The Coronavirus Disease 2019 (COVID-19) pandemic is a syndemic of viral infection and mental health adversity. The pandemic has exacerbated inequalities of access to care in vulnerable populations within the Canadian mental healthcare system. Primary care services are first-line health services in Canada, and are necessary to access specialized services. However, as a result of the limited availability of primary health services, and subsequently, specialized providers (eg, psychiatrists), the demand for these services outweigh the supply. Hitherto, timely access to appropriate services has been cited as a common challenge in Canada as a result of limitations as it relates to resources and in-person activities and support services. While there has been an increase in virtual care opportunities, concerns have been raised with respect to the digital divide. Moreover, while individuals with serious mental illness (SMI) and psychosis are at an increased risk for hospitalization and death from COVID-19, testing and vaccination services have not been prioritized for this population. Taken together, increased funding for mental health service delivery should be emphasized especially for individuals with SMI. There should also be a focus on increased collaboration among individuals with lived experience and health care providers to ensure future policies are developed specifically for this population. Addressing the social determinants of health and prioritizing a continuum of care across various stakeholders may lead to strong integration of care both during and after the pandemic.

## Introduction

The Coronavirus Disease 2019 (COVID-19) pandemic should be characterized as a syndemic of viral infection and mental health adversity.^1^ A confluence of biological and socioeconomic determinants of health, as well as limitations of under resourced and over extended systems, have contributed to COVID-19 vulnerability.^2^ Taken together, the phenotype of an at-risk individual is complex and dynamic.

It is well-established that preexisting mood disorders increase the risk of COVID-19 hospitalization and death.^3^ For example, immune dysregulation, comorbid noncommunicable and communicable diseases, as well as social and economic insecurity (eg, low health literacy, community housing, difficulty accessing preventive health care) have been documented as contributory factors to COVID-19 vulnerability.^4–6^ Individuals with serious mental illness (SMI) and psychosis are at an increased risk for COVID-19 for several reasons, which include but are not limited to, impaired decision-making, ability to adhere to protective measures (eg, social distancing, hand hygiene), and/or comorbid substance use disorders which further confound judgment.^7–9^ They are also more likely to live in congregate housing where social distancing is difficult to achieve. Moreover, the COVID-19 pandemic has exacerbated existing weaknesses in mental healthcare systems (eg, ongoing access to psychiatrists and psychotherapists).^9,10^ Hitherto, individuals with preexisting mood disorders should be recognized as an at-risk group.

The COVID-19 pandemic has underscored and exacerbated the existing vulnerabilities of the Canadian mental healthcare system. Available evidence indicates an increasing proportion of individuals with difficulty accessing mental health resources.^11^ Several factors are attributed to the worsening crisis, which include but are not limited to, public health distancing guidelines, limited healthcare capacity, and under resourced institutions.^11,12^ Taken together, there is a need to identify recommendations for improvements in the Canadian mental healthcare system, especially as it relates to individuals with serious mental illness (SMI).

## Overview of the Canadian Healthcare System

The Canadian healthcare system is founded on a publicly funded structure individually delivered through 10 provincial and 3 territorial health systems.^13^ Canadian healthcare is often characterized as “universal health care.” ^13^ More specifically, services that are deemed medically necessary are provided on a prepaid basis, while procedures that fall outside of this bracket must be paid out-of-pocket or through a third-party insurance provider.^13^ As such, individual provinces and territories are responsible for determining health expenditures that are publicly funded.^13^

Notwithstanding the universal health care system, Canada does not have “universal pharmacare.” ^14,15^ The only medication that is prescribed and administered from Canadian hospitals may be provided to patients at no additional charge.^14,15^ Drugs dispensed outside of these institutions must be paid out-of-pocket or through a third-party insurance provider.^4,15^ However, certain individuals may qualify for publicly funded drug programs. For example, the Ontario Drug Benefit (ODB) program, targeted for Ontarians ages 65 years and older, populations with particular special services and/or individuals who qualify for the Trillium Drug Program which is aimed for individuals with a lower household income relative to drug costs, will be eligible for certain publicly funded medication.^16^ For example, ODB covers 8 out of 10 atypical antipsychotics marketed in Canada.^14,15^

## Challenges Within the Canadian Healthcare System

Primary health care services (eg, basic emergency services, health promotion, preventative treatment) function as first-line health services, as well as a path for accessing specialized services.^13^ For example, individuals seeking a specialized mental healthcare provider must first be referred by a primary healthcare provider.^13^ However, a notable proportion of Canadians with SMI do not have access to a primary care provider. Additionally, psychiatrists in Canada tend to operate as consultants, and do not function as the most responsible physician (MRP). Hitherto, there is a shortage of psychiatrists that are willing and able to provide care for individuals with SMI and psychosis.

Accessing appropriate and timely mental health services has been cited as a common challenge for Canadians. Approximately 78.2% of Canadians have indicated that timely access was attributed to several factors including, but not limited to, difficulty navigating the system to access the appropriate resources, as well as unaffordability of services.^17^ For example, it was previously reported that 30% of Canadians pay out-of-pocket for private practice psychotherapists which cost $950 million annually.^11^

In addition to personal circumstances, barriers in accessing mental health services also include stigma and long wait times. For example, it is not uncommon to wait approximately 6 months to 1 year to receive counseling and therapy in Ontario.^11^ Long wait times may be attributed to under funded community mental health services.^11^ Additionally, allied health professionals (eg, psychotherapists), which may not be covered under universal health coverage, have led to long wait times to see publicly funded healthcare practitioners.^18^ Individuals receiving long acting injectables (LAIs) have faced several barriers to use. Extant literature indicates that stigma, limited knowledge of LAI options, higher cost and logistical challenges of therapy (eg, frequency of visits) have contributed to discontinuation.^19,20^ Taken together, long wait times and limited health resources and government service integration have severely limited mental health access in Canada.

## Challenges Experienced During COVID-19

Data from the 2019 and 2020 Canadian Community Health Survey suggest a decline in mental health following the pandemic. There was a slight decrease in positive mental health from 2019 to 2020 of which this effect was most pronounced among females (ie, 63.0%–59.9%).^12^ Accordingly, there was a slight increase in the proportion of females reporting fair/poor mental health (ie, 9.1% [2019] to 11.7% [2020]). This increase was also observed among Canadians aged 35–49 years (ie, 8.2% [2019], 11.1% [2020]) and 50–64 years (ie, 7.0% [2019] to 9.7% [2020]).

According to results from the Survey on Access to Health Care and Pharmaceuticals during the Pandemic, approximately half of Canadian adults reported difficulties accessing health services; challenges accessing services range from long wait times and appointment availability to closures, and lack of available services and referrals.^21^ For example, across the provinces, 45% of adults in Quebec and 60% in Newfoundland and Labrador expressed concerns regarding health care service access. Approximately 28% of adults who attempted to receive health services reported changes to their original appointment times and/or cancelations as a result of the pandemic.

LAI must be administered by a trained healthcare professional in Canada.^19^ However, as a result of COVID-19, there was increased variability of LAI delivery due to several factors, which include but are not limited to, restricted clinic availability, limitations for caregiver accompaniment, more home visits and interactions with unfamiliar provider(s), and decreased interaction with clinical support staff.^19^ Earlier in the pandemic, the Canadian Psychiatric Association (CPA) issued guidelines emphasizing the importance of the continuation of care of LAI treatment.^19^ More specifically, the risk of discontinuation was perceived to be a greater risk than the potential risk of COVID-19 infection.^19^ Concerns related to the access of LAI treatment as a result of supply chain limitations were reported. However, it is unclear whether these challenges impacted prescription and administration as there was a steady continuation of individuals starting and discontinuing LAIs during the pandemic.

As a result of public health guidelines, a majority of health services that may be performed remotely have shifted to a virtual platform. To increase the capacity for virtual care during the pandemic, it is recommended for offices and providers to identify additional resources (eg, equipment, staff) to ensure that high patient volume is met and to prevent workplace burnout.^22^ However, telemedicine has also introduced access challenges (eg, obtaining appropriate technology, establishment of necessary internet infrastructure, computer literacy, cost of technology).^23^ Furthermore, the limitation of in-person services such as peer support services and engaging lived experience has further exacerbated the loneliness epidemic.^24,25^ Peer support services are an important pillar of well-being for individuals with SMI and psychosis. A survey by the Mental Health Commission of Canada (MHCC) highlighted that 43% of their respondents indicated that the shift to virtual care has furthered the digital divide.^26^

## Testing and Vaccination

Testing and vaccination eligibility have been variable due to the intrinsic health infrastructure in Canada and availability of resources.^27,28^ For example, current COVID-19 testing guidelines in Canada only allow symptomatic and asymptomatic individuals in high-risk settings or high-risk individuals to receive testing. Individuals with symptoms akin to COVID-19 should assume a positive case, isolate and self-monitor. Taken together, individuals outside of high-risk settings and/or situations are not eligible for publicly funded testing.

During the initial administration of vaccines, individuals who were immunocompromised (eg, transplant patients, cancer patients receiving stable active treatment, individuals on dialysis) were prioritized. Individuals with SMI and psychosis were not included in this characterization. However, individuals with enduring neuropsychiatric manifestations are at a higher risk of COVID-19 infection, hospitalization and death, and should be prioritized for booster shots and testing.

## Recommendations

Given the need for health services during the pandemic, the Canadian government has set out various policies to adapt to the growing demand.^22^ For example, there has been an expansion of responsibilities among health professionals (eg, prescribing authority, retirees). Furthermore, hospitals have been notified of potential crowding and as such, to mitigate the impact of high demand on hospital resources, recommendations have been made to ensure that there is a stockpile of essential resources (eg, medications), and training and administration of health care providers in disparate roles have been established. Taken together, policies have focused on the maximization and augmentation of current resources as well as improvements to optimize efficiency in care and resource allocation.^22^ Moreover, the Canadian government has committed $500 million in funding towards mental health, substance use, and homelessness challenges during COVID-19.^22,29^ Additionally, the federal government has implemented e-mental health initiatives such as Wellness Together Canada as a mental health support.^30^

The burden of mental illness (eg, direct health care costs, loss of productivity, reduced quality of life) is estimated to be $51 billion.^11^ According to the Survey on Access to Health Care and Pharmaceuticals during the Pandemic, approximately half of Canadians who attempted to access mental health care services during the first year of the pandemic experienced challenges.^21^

Increasing funding in mental health service delivery (eg, psychologists, counselors, community-based services) will likely decrease wait times and reduce the need for private expenditures. Additionally, various healthcare stakeholders (eg, researchers, funders) should work together to efficiently identify and establish a shared policy agenda for individuals with SMI.^31^ There is also a need to publish lived experiences from this population in an effort to better understand the experiences and challenges during COVID-19, as well as form meaningful collaborations in an effort to develop tailored policies at a community, provincial and federal level.^31^

Additionally, there is a need to address social determinants of health such as safe living and housing conditions and community-based support programs to increase social support networks. Accordingly, decreasing the digital divide will increase the utilization of virtual care services as well as improve the integration of existing community programs into a digital platform. Furthermore, ongoing government support should be allocated towards services that satisfy basic human needs (eg, food banks, employment opportunities). Moreover, testing and vaccination access should be prioritized for individuals with SMI and psychosis.

Prioritizing a continuum of care by leveraging virtual services in a patient-centric approach will require cooperation of various stakeholders (eg, community, government, patients). A formal system should be implemented to ensure the education of available support tools and technology to ensure ease of access and a greater return on investment. These programs should focus on strong integration to ensure an appropriate transition to and from in-person care, and regular follow-up.

Taken together, the COVID-19 pandemic has highlighted weaknesses within Canada’s health systems and the preexisting inequities that stratify vulnerable groups. Prioritizing funding and resources into the implementation of services and tools to reduce barriers to accessing mental health care is necessary and will continue to be essential after the pandemic.
